# Assessment of the Vitamin B12 Status of Pregnant Women in Nigeria Using Plasma Holotranscobalamin

**DOI:** 10.5402/2011/365894

**Published:** 2011-07-14

**Authors:** Dorothy J. VanderJagt, Innocent A. O. Ujah, Eugene I. Ikeh, Jessica Bryant, Victor Pam, Amelia Hilgart, Michael J. Crossey, Robert H. Glew

**Affiliations:** ^1^Department of Biochemistry and Molecular Biology, University of New Mexico Health Sciences Center, MSC08 4670, Albuquerque, NM 87131-0001, USA; ^2^Department of Obstetrics and Gynaecology, Jos University Teaching Hospital, Jos, PMB 2076, Nigeria; ^3^Department of Medical Microbiology, University of Jos, Jos, PMB 2076, Nigeria; ^4^TriCore Reference Laboratories, Albuquerque, NM 87102, USA

## Abstract

Maternal vitamin B12 deficiency during pregnancy is an independent risk factor for neural tube defects and other neurological problems in infants. We determined the vitamin B12 status of 143 pregnant women in Nigeria representing all trimesters who presented to an antenatal clinic in Jos, Nigeria, using holotranscobalamin II levels (holoTCII), which is a measure of the vitamin B12 that is available for uptake into tissues. The holoTCII concentration ranged from 13 to 128 pmol/L. Using a cutoff of 40 pmol/L, 36% of the women were classified as vitamin B12-deficient. HoloTCII concentrations correlated negatively with plasma homocysteine levels (*r* = −0.24, *P * = 0.003) and positively with red blood cell folate concentrations (*r* = 0.28, *P* < 0.001). These data underscore the importance of supplementing pregnant women in Nigeria with vitamin B12 in order to ensure adequate vitamin B12 status and decrease the risk for neural tube defects.

## 1. Introduction

Adequate vitamin B12 status during pregnancy is critical since maternal vitamin B12 deficiency is associated with increased risk for several adverse pregnancy outcomes for both mother and fetus. These risks include neural tube defects [1–4], intrauterine growth retardation [5], preeclampsia [6] and early miscarriage [7, 8]. Furthermore, fetal vitamin B12 accumulation during gestation is the major determinant of the B12 status of the newborn and of stores in infancy [9–12]. Even though a mother may not exhibit any hematological or neurological symptoms of B12 deficiency, an exclusively breast-fed infant born to a mother who is deficient in vitamin B12 can develop symptoms within several months following delivery [13, 14]. These include failure-to-thrive, megaloblastic anemia, and neurological symptoms [15]. 

Vitamin B12 is present only in foods of animal origin such as meat, dairy products, and fish [16, 17]. Thus, individuals adhering to a vegetarian diet or residing in parts of the developing world where consumption of foods from animal sources is limited because of cost or availability will be predisposed to vitamin B12 deficiency [18]. There is increasing evidence that the global prevalence of vitamin B12 deficiency, particularly in the developing parts of the world, is underappreciated [11, 19–22]. 

In a previous study of 146 pregnant women in Nigeria [23], we found that 9% of them had a serum vitamin B12 concentration below 148 pmol/L, which is the cutoff for B12 deficiency [24]. In a subsequent study of 98 pregnant Nigerian women [25], we documented that 12% of the subjects had vitamin B12 levels in the deficiency range and about one in five had an elevated serum methylmalonic (MMA) concentration, which is a specific metabolic marker of vitamin B12 deficiency [26]. Applying the criteria proposed by Carmel and associates [24] of a combined low vitamin B12 level (<148 pmol/L) and a MMA concentration greater than 270 *μ*mol/L, 8% of the women in that study were determined to have subclinical vitamin B12 deficiency. 

Measurement of the total vitamin B12 concentration in plasma is the usual method for assessing vitamin B12 status. However, neurological and hematological symptoms of deficiency can occur in individuals with plasma vitamin B12 concentrations in the low-normal range [27]. Conversely, some individuals with low vitamin B12 concentrations remain symptom-free. Moreover, the plasma vitamin B12 concentration is not a reliable indicator of vitamin B12 status in pregnancy since there is a gradual, physiologically normal decline in the plasma concentration of vitamin B12 during an uncomplicated pregnancy [28, 29]. This decline is thought to be due to hemodilution, hormonal changes, alterations in the concentration of vitamin B12 binding proteins, or active transport of vitamin B12 across the placenta [30]. 

In the circulation, vitamin B12 is bound by two different transport proteins: approximately 70%–80% is carried by haptocorrin while the remainder is bound to transcobalamin II (holotranscobalamin, holoTC) [31]. Only that vitamin B12 which is bound to holoTC is available to tissues through uptake mediated by receptors for the B12/transcobalamin complex [32]. The function of circulating vitamin B12 bound to haptocorrin is not known, but may represent that portion of vitamin B12 which is being returned to the liver for excretion in the bile or physiologically inert metabolites of vitamin B12. 

Holotranscobalamin is a more sensitive indicator of vitamin B12 status than the total serum vitamin B12 level or the serum concentration of MMA and homocysteine (Hcys), both of which are elevated in vitamin B12 deficiency [33–35]. Moreover, in an investigation of the circadian variation of holoTC, Hvas and coworkers [36] found no significant changes in serum holoTC concentrations in healthy vitamin B12-replete subjects consuming a standard diet. They concluded, therefore, that holoTC is not an indicator of recent dietary intake but rather a marker of long-term vitamin B12 status. In addition, in a longitudinal study of healthy pregnant women from 18 weeks gestation up to the time of delivery, Morkbak and coworkers [37] observed that although serum vitamin B12 levels decreased over the course of the pregnancy, holoTC levels remained constant in women with an adequate intake of vitamin B12. 

In light of the importance of adequate vitamin B12 status to the well-being of the fetus during pregnancy, the present study was designed to use the measurement of plasma holoTC to assess the vitamin B12 status of pregnant women attending at an antenatal clinic in Nigeria for the first time in their current pregnancy.

## 2. Subjects and Methods

### 2.1. Subjects

 This study was conducted at the Antenatal Clinic of the Jos University Teaching Hospital (JUTH), a tertiary care facility in Jos, Nigeria. Healthy women representing all trimesters who presented at the clinic for the first time during their current pregnancy were invited to enroll in the study. Subjects were enrolled after the purpose and requirements of the study had been explained to them in English or Hausa by the attending physician in the clinic. Informed written consent was obtained from each participant. This study was approved by the Ethics Review Committee of JUTH and by the Human Research Review Committee of the University of New Mexico Health Sciences Center, Albuquerque, New Mexico. 

Self-reported information regarding subjects was captured by interview and included age, gravidity, parity, occupation, educational background, ethnic group, previous clinic visits, vitamin supplementation, and fever. Gestational age was estimated based on the date of the last menstrual cycle. At the completion of the interview, subjects were asked to provide a blood sample for biochemical analyses and malaria testing. Since infection with intestinal parasites is known to affect vitamin B12 status [38, 39], each subject was given a container and asked to return with a stool sample the next day for the analysis of the presence of intestinal parasites. 

### 2.2. Anthropometric Measurements

The weight of each subject was measured using a portable scale accurate to 0.5 kg that was standardized each day using a known weight. Height was measured using a portable stadiometer readable to 0.25 cm (Creative Health Products, Plymouth, Mich, USA).

### 2.3. Malaria Screening

Infection with malaria was determined using the *CareStart *Malaria pLDH rapid test (Access Bio, Inc., NJ, USA). The test, which is designed for the differential diagnosis of *Plasmodium falciparum* and other *Plasmodium *species, has a sensitivity of 96% and specificity of 98.5% when compared to microscopic examination of whole blood. 

### 2.4. Intestinal Parasite Screening

 Stool samples were preserved in 10% (v/v) formol-saline and later screened for intestinal parasites using a formol-ether concentration method [40]. Briefly, approximately 2 g of stool sample were placed in a 15 mL conical centrifuge tube and brought to 7 mL with 10% (v/v) formol-saline. Ether (3 mL) was then added to give a total volume of 10 mL. The sample was vortexed and then centrifuged at 1200 ×g for 5 min in a clinical centrifuge. The supernatant was decanted, and one drop of the pellet was added to a glass slide and stained with Lugol's iodine and then examined under a light microscope for the presence of parasites [41]. 

### 2.5. Biochemical Measurements

A blood sample was obtained from each subject by venipuncture and collected in a heparinized vacutainer tube. The packed cell volume (PCV) was determined for each subject by centrifugation of an aliquot of whole blood in a microhaematocrit tube in a READACRIT centrifuge (Clay Adams, Parsippany, NJ, USA) for 5 min. The remaining sample was divided into two portions: 1 mL whole blood to be used for the determination of hemoglobin and red blood cell folate was transferred to a cryovial tube and stored at −70°C; the remainder of the blood sample was centrifuged at 1200 ×g to separate plasma and cells and the plasma fraction was stored at −70°C. The samples were transported in the frozen state to Albuquerque, NM for biochemical analyses. 

Hemoglobin concentration was determined by colorimetric analysis of lysed whole blood using the Hemoglobin *B* test kit (Wako Chemicals, Richmond, VA, USA). Total vitamin B12 was measured using the IMMULITE 2500 Vitamin B12 solid phase, two-site chemiluminescent enzyme immunoassay in the IMMULITE 2500 analyzer (Diagnostic Products Corporation, Los Angeles, Calif, USA). The test is sensitive to 92 pmol/L and has a coefficient of variation (CV) less than 10%. Vitamin B12 bound to transcobalamin II (holoTC) was determined using the AxSYM Active B12 microparticle enzyme immunoassay (Abbott Diagnostics, AxSYM, Abbott Park, Ill, USA). The test is sensitive to 1 pmol/L with a coefficient of variation of less than 10%. 

Plasma folate and whole blood folate concentrations were determined using the IMMULITE 2500 Folic Acid Assay for the IMMULITE 2500 analyzer (Diagnostic Products Corporation, Los Angeles, Calif, USA). The red blood cell folate concentration was calculated using the whole blood folate concentration, hematocrit and plasma folate concentration. Plasma homocysteine (Hcys) was measured using the IMMULITE 2000 Homocysteine Competitive Immunoassay. Plasma ferritin was measured using the VITROS Ferritin two-step immunometric assay on the VITROS Eci Immunodiagnostic System (Ortho-Clinical Diagnostics, Inc., Rochester, NY, USA). 

### 2.6. Statistical Analysis

Descriptive statistics, group comparisons, and correlation analyses were performed using the Number Cruncher Statistical Software (NCSS, Kaysville, Utah, 2006). Variables with a normal distribution are presented as the mean ± 1 standard deviation. Variables that were not normally distributed are presented as the median (minimum-maximum). Comparisons between trimesters were performed using one-way ANOVA. The vitamin B12 concentrations for the women with intestinal parasites were compared to those without intestinal parasites using the *t*-test. Correlations were determined using the Spearman correlation coefficient. A *P* value of ≤0.05 was considered statistically significant. 

## 3. Results

### 3.1. Subjects

 A total of 143 women were enrolled in the study. The women ranged in age from 18 to 53 years (Table 1). The majority of the subjects were Hausa (*n* = 35), followed by Berom (*n* = 18), Fulani (*n* = 12), Igbo (*n* = 9), and Yoruba (*n* = 7). The remainder (*n* = 61) reported other ethnic designations. Forty-three of the women reported having less than six years of schooling, 50 had greater than six years of school, and 48 had attended university. The educational background of two women was not available. Housewives accounted for the highest number of subjects (*n* = 38), followed by tailors (*n* = 31), students (*n* = 17), traders (*n* = 16), professionals (*n* = 12), teachers (*n* = 10), clerical workers (*n* = 10), and other occupations (*n* = 8). 

The majority of the women (*n* = 88, 61%) were in the second trimester; 30 (21%) were in the first trimester and 25 (17%) in the third trimester. Supplements were used by 11 women: 10 were taking iron and folate supplements, whereas only 1 subject reported taking a folate supplement only. Eight of the 11 women who were taking supplements had previous pregnancies. The mean blood pressure for all of the subjects was in the normal range (Table 1), except for one woman who had an elevated blood pressure (>140/>90 mmHg); she was evaluated and treated by a physician. 

### 3.2. Malaria Screening

 Six of the subjects tested positive for malaria; however, only one woman reported experiencing fever. All positive results were due to infection with *P. falciparum*, and all women who tested positive for malaria were given medication. 

### 3.3. Stool Screening

Stool samples were obtained from 121 of the 143 subjects. At least one species of intestinal parasite was found in the stool samples of 29 of the 121 subjects. Sixteen of the women tested positive for more than one parasite. The most common parasite found was *Entamoeba histolytica* (*n* = 18), followed by *Endolimax nana *(*n* = 12), *Entamoeba coli* (*n* = 12), *Dicrocoelium dendriticum* (*n* = 3), hookworm (*n* = 2), and *Giardia lamblia *(*n* = 2).

### 3.4. Biochemical Analyses

The results of the biochemical analyses are summarized in Table 2. The mean hemoglobin concentration for the 143 subjects was 108 g/L. According to the Centers for Disease Control and Prevention [42], a hemoglobin concentration less than 110 g/L in the first and third trimesters and less than 105 g/L in the second trimester is indicative of anemia. According to these criteria, 56 (36%) of the subjects were anemic: 5 of the 30 subjects in the first trimester, 33 of the 88 women in the second trimester, and 9 of 25 women in the third trimester. None of the subjects in the anemic category reported taking an iron or folic acid supplement. Despite the high number of subjects in the anemic range, only 16 (11%) of them had a ferritin concentration below 10 ng/mL [43].

Five (4%) of the total subjects had a serum folate concentration less than 7.7 nmol/L, whereas 117 (81%) had a red blood cell folate concentration below 317 nmo/L. Although serum folate concentrations did not correlate with plasma Hcys levels, there was a significant negative correlation between plasma Hcys concentrations and red blood cell folate concentrations (*P* = 0.015, *r* = −0.20). 

**Figure 1:**
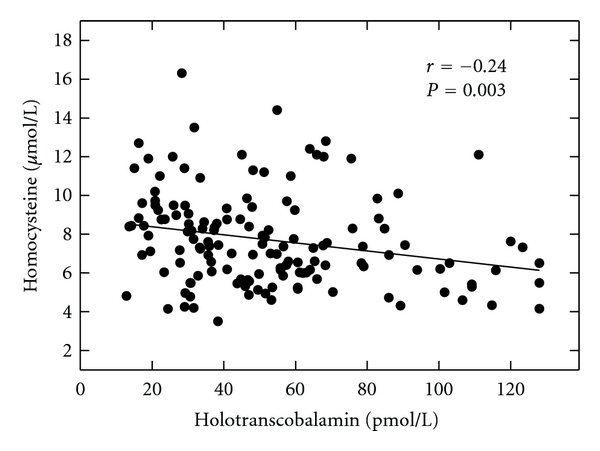
The relation between serum holotranscobalamin II concentrations and serum homocysteine concentrations; *r* = −0.24, *P* = 0.003.

Thirty-six (25%) of the pregnant women had a plasma total vitamin B12 concentration below 148 pmol/L, the cutoff for vitamin B12 deficiency: 27% of women in the first trimester, 23% in the second trimester, and 32% in the third trimester. The plasma vitamin B12 concentrations of the subjects in the second and third trimesters were significantly lower than the values of subjects in the first trimester (*P* = 0.02, Table 2). The plasma vitamin B12 concentration was positively correlated with red blood cell folate level (Figure 1, *r* = 0.28, *P* < 0.001) and negatively correlated serum Hcys concentration (*r* = −0.24, *P* = 0.003). 

**Figure 2:**
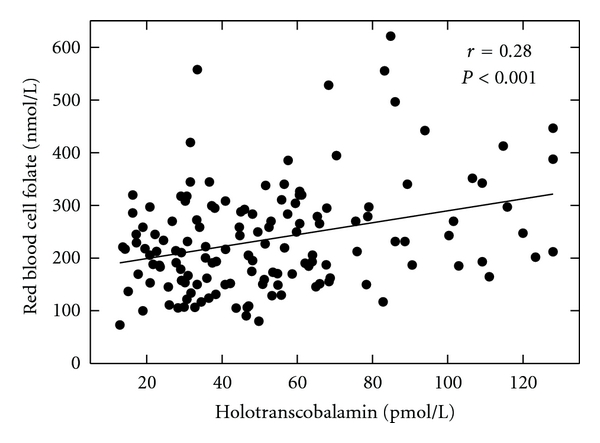
The relation between serum holotranscobalamin II concentrations and red blood cell folate concentrations.; *r* = 0.28; *P* < 0.001.

The holoTC concentrations for the pregnant women ranged from 13 to 128 pmol/L, with a mean value of 53 pmol/L. Using a holoTC cut-off value of 40 pmol/L determined for the AxSYM Active B12 immunoassay [44], 52 (36%) subjects were vitamin B12 deficient: 30% of the women in the first trimester, 38% in the second trimester, and 40% in the third trimester. The holoTC levels were not different between trimesters. Twenty-eight of the 56 women who were classified as anemic on the basis of hemoglobin concentration had a holoTC level below the 40 pmol/L cutoff. Holotranscobalamin II concentration was negatively correlated with serum Hcys level (*r* = −0.24, *P* = 0.003, Figure 1), but positively correlated with red blood cell folate concentration (*r* = 0.28, *P* < 0.001; Figure 2). Eight of the 29 women with intestinal parasites had a plasma transcobalamin concentration in the deficient range.

## 4. Discussion

Inadequate maternal vitamin B12 status during pregnancy places the fetus at risk for a neural tube defect before birth, and for anemia and neurological disorders following birth. Using the plasma holoTC concentration as an indicator of physiologically available vitamin B12, in the present study we found that 36% of pregnant Nigerian women presenting at an antenatal clinic for the first time in their current pregnancy had a plasma holoTC concentration indicative of vitamin B12 deficiency.

Vitamin B12 accumulation *in utero* is the major determinant of vitamin B12 status in the newborn and throughout infancy (9–12). Vitamin B12 is actively transported across the placenta [45], and strong correlations between maternal and newborn vitamin B12 status have been documented [46, 47]. Giugliani and associates [45] estimated that an infant born to a vitamin B12-replete mother will have vitamin B12 stores in the 25 to 30 *μ*g range. Based on an average breast milk concentration of 0.42 *μ*g/L in B12-replete mothers [48], an infant consuming 780 mL breast milk would obtain approximately 0.3 *μ*g/day of vitamin B12 from milk. This amount is close to the recommended adequate intake (AI) of 0.4 *μ*g/day for infants up to 6 months of age [49]. The remaining vitamin B12 must be supplied by the hepatic vitamin B12 stores of the infant. Assuming a withdrawal of 0.10 to 0.20 *μ*g/day from hepatic stores, an infant would normally have a 4- to 6-month reserve of vitamin B12. However, the vitamin B12 content of milk from women who have inadequate vitamin B12 intake will be lower, in the range of 0.13 to 0.36 *μ*g/L [50, 51], and the vitamin B12 stores of an infant born to such a mother would be expected to be correspondingly low. 

Although adults can tolerate inadequate intake of vitamin B12 for a long period of time, infants on the other hand develop symptoms of B12 deficiency much more rapidly if they have inadequate liver stores of the vitamin. Symptoms of vitamin B12 deficiency in infants include failure-to-thrive, irritability, apathy, anorexia, refusal of solid foods, megaloblastic anemia, and developmental delays [15]. Although treatment with vitamin B12 can reverse these symptoms, infants treated for vitamin B12 deficiency often suffer long-term cognitive and developmental retardation [52] depending on the severity and duration of the vitamin deficiency. 

The basis for the neurological effects of vitamin B12 deficiency in infants has not been established, but several mechanisms have been proposed [53]. These include delayed myelination or demyelination of nerves, an alteration in the S-adenosylmethionine-S-adenosylhomocysteine ratio (SAM/SAH), and an imbalance in neurotrophic and neurotoxic cytokines. Guerra-Shinohara and coworkers [54] reported that cord blood from newborns of mothers with low levels of serum vitamin B12 had below-normal concentrations of SAM and elevated concentrations of SAH, and concluded that methylation reactions were impaired in these newborns. Therefore, the accumulation of odd-chain fatty acids and impaired methylation processes could contribute to the synthesis of unstable myelin and subsequent demyelination of nerves. 

Folic acid fortification of food in several countries, including the US and Canada, has been proven to greatly reduce, but not eliminate entirely, the incidence of NTDs [55]. Other factors, including vitamin B12, have been shown to be independent risk factors for NTDs [1–4]. The reported association between maternal vitamin B12 insufficiency and risk for neural tube defects is of special concern for pregnant women in sub-Saharan Africa because of the high incidence of NTDs. Although there are few reports documenting the incidence of neural tube defects in Nigeria and other regions of sub-Saharan Africa, a three-year prospective study in Jos, Nigeria recorded an incidence of 7/1000 deliveries [56]. In a more recent three-year retrospective study in southern Nigeria, Ugwu and coworkers [57] found the incidence of NTDs to be 0.95/1000 births. In neighboring Cameroon, an NTD incidence of 1.99/1000 births was reported over a 10-year period [58]. In contrast, the incidence of neural tube defects for the Group 8 (G8) countries has been reported to be much lower at 0.3 to 0.4/1000 births [59]. 

Red-cell folate is regarded as a better indication of tissue folate content than the serum folate level. The correlation between holoTC and red-cell folate concentration we observed in the present study underscores the importance of vitamin B12 status in the maintenance of tissue folate [60]. 5-Methyltetrahydrofoly monoglutamate is the principle form of folate in blood and the form taken by tissues. Once inside the cell, the monoglutamate form must be elongated to the polyglutamate form in order to be retained by the cell. However, 5-methyltetrahydrofoalte is a poor substrate for polyglutamate synthase, the enzyme that catalyzes this polymerization reaction, and must first be converted to tetrahydrofolate in the vitamin B12-dependent reaction catalyzed by methionine synthase. Therefore, in vitamin B12 deficiency, the concentration of tissue folate polyglutamate is commonly decreased while the 5-methyltetrahydrofolate form in serum is elevated. In the present study, 45/117 (38%) of the subjects with low red blood cell folate had a plasma holoTC concentration below 40 pmol/L, whereas only 5 (4%) of the subjects had a plasma folate concentration below the lower limit of normal (7.7 nmol/L). 

One of the limitations of the present study was that dietary intake of vitamin B12 was not assessed. A previous study of the diets of individuals residing in the same locality as the present study found that the dietary intake of vitamin B12 by non-pregnant women was 2.9 ± 1.7 *μ*g/day [61]. The current Adequate Intake (AI) for pregnant women set by the Institute of Medicine in the US is 2.6 *μ*g/day [62]. However, in a recent study of the relation between dietary vitamin B12 intake and B12-related biomarkers in a healthy population between 18–50 years of age, Bor and coworkers [63] found that all of the biomarkers used, including plasma vitamin B12, holoTC, MMA, and Hcys, leveled off at a daily intake between 4.2 and 7.0 *μ*g/day. They suggested that the current AI for vitamin B12 may be inadequate even for a healthy population. In a study of postmenopausal Danish women, an intake of 6 *μ*g/day was determined to normalize all vitamin B12-related parameters [64]. 

The dietary source of vitamin B12 strongly influences its bioavailability. In a study of the sources of vitamin B12 and their association with plasma vitamin B12 levels, Vogiatzoglou and coworkers determined that the bioavailability of vitamin B12 was greater from dairy products and fish than from meat [65]. In the dietary study we conducted in Jos, Nigeria [63], only 10% of the protein in the diets of the women was derived from eggs and milk, with 25% of protein coming from meat. 

Women attending the antenatal clinics in this region are routinely given iron and folate supplements when they register at the clinics in order to prevent anemia, but are not prescribed multivitamins or vitamin B12 supplements. The present study provides reason to suggest that pregnant women attending antenatal clinics in Nigeria would benefit from vitamin B12 supplementation. This practice would support the Millenium Development Goals [66] to promote the health of mothers and infants in developing countries. 

## Figures and Tables

**Table 1:** Summary of the anthropometric characteristics of pregnant women attending an antenatal clinic in Jos, Nigeria, for the first time in their current pregnancy.

	Total		Trimester	
		1	2	3
	(*n* = 143)	(*n* = 30)	(*n* = 88)	(*n* = 25)
Parameter	Mean ± S.D.			

Age (yrs)	28.0 ± 6.1	26.9 ± 5.9	28.1 ± 5.9	29.1 ± 6.7
Weight (kg)	63.3 ± 13.2	61.4 ± 12.8	63.1 ± 13.1	66.5 ± 14.2
Height (cm)	159.8 ± 5.9	160.2 ± 5.1	160.1 ± 6.1	157.9 ± 5.8
Gravida (*n*)	3 (1–10)*	3 (1–7)	2 (1–10)	5 (1–9)
Blood pressure (mmHg)				
Systolic	109 ± 15	110 ± 13	111 ± 16	105 ± 15
Diastolic	64 ± 10	66 ± 9	64 ± 11	60 ± 10

* Median (minimum-maximum).

**Table 2:** Summary of the biochemical analyses for pregnant women attending an antenatal clinic in Jos, Nigeria, for the first time in their current pregnancy.

	Total		Trimester	
		1	2	3
	(*n* = 143)	(*n* = 30)	(*n* = 88)	(*n* = 25)
	Mean ± SD			

Hemoglobin (g/L)	108 ± 13	114 ± 11^a^	107 ± 13	106 ± 14
Hematocrit (%)	37 ± 4	39 ± 4^b^	36 ± 4	36 ± 4
Vitamin B12 (pmol/L)	199 (111–738)*	346 (111–738)^c^	197 (111–738)	164 (111–738)
Holotranscobalamin (pmol/L)	53 ± 28	52 (14–123)	52 (15–128)	45 (13–128)
Serum folate (nmol/L)	20 ± 12	27 ± 12^e^	19 ± 12	14 ± 5
Red blood cell folate (nmol/L)	240 ± 110	271 ± 132	241 ± 106	199 ± 86
Homocysteine (*μ*mol/L)	7.7 ± 2.4	7.7 ± 2.1	7.7 ± 2.4	7.6 ± 2.9
Ferritin (ng/mL)	27.5 (4.8–285)	55.6 (12–218)^d^	24.4 (4.8–285)	17.6 (6.8–160)

* Median (minimum-maximum); trimester 1 different from trimesters 2 and 3, ^a^ *P* = 0.016, ^b^ *P* ≤ 0.001, ^c^ *P* = 0.015, ^d^ *P* < 0.001; ^e^significant difference between all trimesters, *P* ≤ 0.001.
